# Clinical Characteristics of Myalgic Encephalomyelitis/Chronic Fatigue Syndrome (ME/CFS) Diagnosed in Patients with Long COVID

**DOI:** 10.3390/medicina58070850

**Published:** 2022-06-25

**Authors:** Kazuki Tokumasu, Hiroyuki Honda, Naruhiko Sunada, Yasue Sakurada, Yui Matsuda, Koichiro Yamamoto, Yasuhiro Nakano, Toru Hasegawa, Yukichika Yamamoto, Yuki Otsuka, Hideharu Hagiya, Hitomi Kataoka, Keigo Ueda, Fumio Otsuka

**Affiliations:** 1 Department of General Medicine, Okayama University Graduate School of Medicine, Dentistry and Pharmaceutical Sciences, Okayama 700-8558, Japan; tokumasu@okayama-u.ac.jp (K.T.); ppgf1hrd@okayama-u.ac.jp (H.H.); naru.kun.red.1117@gmail.com (N.S.); pzaf6h9w@s.okayama-u.ac.jp (Y.S.); m05089ym@gmail.com (Y.M.); pi291nd8@s.okayama-u.ac.jp (K.Y.); me421055@s.okayama-u.ac.jp (Y.N.); t.hase5178.1@gmail.com (T.H.); pk2d6hlo@s.okayama-u.ac.jp (Y.Y.); otsuka@s.okayama-u.ac.jp (Y.O.); hagiya@okayama-u.ac.jp (H.H.); hitomik@md.okayama-u.ac.jp (H.K.); philopotchy@live.jp (K.U.); 2 Clinical & Educational Center for Kampo Medicine, Okayama University Hospital, Okayama 700-8558, Japan

**Keywords:** general fatigue, post-exertional malaise, long COVID, myalgic encephalomyelitis/chronic fatigue syndrome, post COVID-19 condition

## Abstract

*Background and Objectives:* COVID-19 can be serious not only in the acute phase but also after the acute phase and some patients develop ME/CFS. There have been few studies on patients with long COVID in whom ME/CFS was diagnosed by physicians based on standardized criteria after examinations and exclusion diagnosis and not based on only subjective symptoms. The purpose of this study was to elucidate the detailed characteristics of ME/CFS in patients with long COVID. *Materials and Methods*: A retrospective descriptive study was performed for patients who visited a COVID-19 aftercare clinic established in Okayama University Hospital during the period was from February 2021 to April 2022. *Results*: Clinical data were obtained from medical records for 281 patients, and 279 patients who met the definition of long COVID were included. The overall prevalence rate of ME/CFS diagnosed by three sets of ME/CFS criteria (Fukuda, Canadian and IOM criteria) was 16.8% (48.9% in male and 51.1% in females). The most frequent symptoms in ME/CFS patients were general fatigue and post-exertional malaise (89.4% of the patients), headache (34.0%), insomnia (23.4%), dysosmia (21.3%) and dysgeusia (19.1%). Dizziness, chest pain, insomnia and headache were characteristic symptoms related to ME/CFS. The male to female ratio in ME/CFS patients was equal in the present study, although ME/CFS was generally more common in women in previous studies. Given that patients with ME/CFS had more severe conditions in the acute phase of COVID-19, the severity of the acute infectious state might be involved in the pathophysiology of ME/CFS. *Conclusions*: The prevalence rate of ME/CFS and the characteristic sequelae in the long COVID condition were revealed in this study.

## 1. Introduction

The pandemic of novel coronavirus disease 2019 (COVID-19) has been continuing for more than 2 years and more than 285 million cases of infection and 5.4 million deaths have been reported worldwide [1]. Besides the acute-phase symptoms, COVID-19 can also cause prolonged sequelae, which have been termed as long COVID or post-acute sequelae of SARS-CoV-2 infection (PASC) and have recently been defined by WHO as post COVID-19 condition [2,3]. Various symptoms including general malaise, dysosmia, dysgeusia, low-grade fever, headache, and hair loss have been reported in patients with long COVID worldwide [3,4] as well as in Japan [5,6,7]. A meta-analysis of studies on long COVID including one symptom or more showed that 80% of the patients with COVID-19 have long-lasting symptoms, and long COVID is therefore an important clinical problem [4].

In COVID/post COVID-19 conditions, general fatigue and post-exertional malaise (PEM) are the most common sequelae [4,6] and it has been reported that some patients with long COVID develop myalgic encephalomyelitis/chronic fatigue syndrome (ME/CFS) [8]. ME/CFS is a debilitating illness with a wide range of manifestations, and the diagnosis is mainly based on symptoms (fatigue and post-exertional malaise), sleep disturbance, pain and neurological/cognitive dysfunction that have continued for at least six months [9,10,11]. Due to a lack of biological illness markers, there are approximately 20 case definitions for ME/CFS [12,13], with the most frequently utilized case definitions being the Fukuda criteria [9], Canadian Consensus Criteria [10] and Institute of Medicine (IOM) criteria [11]. The intractable syndrome occasionally develops as a severe and long-lasting condition, causing a multifaceted problem [14].

In community-based epidemiological research, the prevalence rate of ME/CFS in adults in the United States has been estimated to be 0.42% [15] and a recent meta-analysis of studies spanning 13 countries estimated a prevalence rate of 0.89% [16]. In a study conducted in Iran in which COVID-19 hospitalized patients were followed for 6 months, the prevalence rate of ME/CFS based on the Fukuda criteria was 17.5% [8]. A larger investigation including 3762 patients (18 years or older) from 56 countries revealed that more than 50% of people with confirmed or suspected COVID-19 complained of lingering symptoms at 3 months after the onset of COIVD-19 [17]. It was estimated that 10% of long COVID patients will be left with an illness that meets the IOM case definition for ME/CFS [18].

Previous studies on the prevalence of ME/CFS after COVID-19 have been conducted by using questionnaire-based surveys [8,17,18]. However, the possibility of underlying physical diseases such as endocrine disorders was not fully ruled out in those studies, and the results were based only on subjective symptoms. There has been no study that focused on characteristics of patients with ME/CFS diagnosed by standardized criteria for the disease and exclusion of other potentially associated diseases by physicians. The purpose of the present study was to determine the prevalence of ME/CFS and the clinical backgrounds of patients with ME/CFS caused by post COVID-19 conditions.

## 2. Patients and Methods

### 2.1. Enrollment of Patients

This study was a descriptive study conducted in a single facility. A COVID-19 aftercare clinic (CAC) was established on 15 February 2021 in the Department of General Medicine, Okayama University Hospital, a tertiary hospital with 865 beds located in the western area of Japan. The purpose of establishing the CAC was to evaluate and manage patients who have suffered from post COVID-19 condition symptoms for more than one month (four weeks) after the onset of COVID-19. Most of the consulting patients were referred from outside medical facilities.

### 2.2. Collection of Clinical Data

Clinical information on patients who visited the CAC was obtained retrospectively. Medical records for 281 patients during the period from February 2021 to April 2022 were carefully reviewed. Long COVID was defined as symptoms that persist for more than one month after the onset of COVID-19 [2,3,19]. Information on age, gender, body mass index (BMI), severity of the acute phase of COVID-19, duration after the onset of COVID-19 to visiting the CAC, history of COVID-19 vaccination, and clinical symptoms of long COVID was obtained from medical records. Through physician’s face-to-face examinations, all of the patients were investigated for any underlying diseases. Endocrine and metabolic disorders, such as pituitary disorders, thyroid disorders, adrenal dysfunction, hyponatremia and hypercalcemia, were scrutinized as necessary. We assessed Fatigue Assessment Scale (FAS) [20], EuroQol 5 dimensions 5-level (EQ-5D) [21] and Self-Rating Depression Scale (SDS) [22,23] using patient-reported questionnaires at the first visit. Regarding the criteria of ME/CFS, we used three internationally standardized sets of ME/CSF criteria for the eligible patients: the Fukuda criteria [9], the Canadian Consensus Criteria (CCC) [10], and the IOM criteria [11]. Between late April and mid-May 2022, the co-authors shared the task of reviewing all patient medical record data to check whether patients met each of the three criteria for ME/CFS. As a reconfirmation, the principal researcher and outpatient physicians who managed the patients reviewed the medical record data for patients who met each of the three criteria for ME/CFS. This process was used to establish the ME/CFS group.

### 2.3. Statistical Analyses

All statistical analyses were performed using Stata/SE 17.0 (StataCorp, 4905 Lakeway Dr, College Station, TX, USA). The characteristics of ME/CFS patients and non-ME/CFS patients were compared using Mann–Whitney U-tests for continuous variables and Fisher’s exact tests or Pearson’s χ^2^ tests for categorical variables. A *p* value of less than 0.05 was considered statistically significant.

### 2.4. Ethical Approval

Information regarding the present study was provided on our hospital wall and on the website of our hospital, and patients who wished to opt out were offered that opportunity. Informed consent from the patients was not necessary due to the anonymization of data. This study was approved by the Ethics Committee of Okayama University Hospital (No. 2105-030) on 28 April 2022 and adhered to the Declaration of Helsinki.

## 3. Results

Of the 281 patients who were enrolled, we excluded two patients because they visited our CAC in less than 4 weeks after the onset of COVID-19, and data for 279 patients visiting our CAC were obtained. There were no patients with obvious endocrine or metabolic abnormalities. The percentages of patients meeting the three sets of criteria were 17.2% for Fukuda Criteria, 17.9% for CCC, and 17.9% for IOM criteria (Table 1). Forty-seven patients (16.8%) met all of the three sets of criteria.

The clinical backgrounds of the post COVID-19 patients with ME/CFS and the post COVID-19 patients without ME/CFS are shown in Table 2. The median ages of patients in the two groups were 40 years and 41 years, respectively, without an apparent deviation in age distribution. The ME/CFS patients included 23 males (48.9%) and 24 females (51.1%), and the percentages were similar in the non-ME/CFS patients. BMIs and percentages of patients with a smoking habit and alcohol drinking habit were also not significantly different between the two groups. Frequency of hospitalization during the acute phase was significantly higher in ME/CFS patients (44.7% vs. 27.6%; *p* = 0.02), while the percentages of patients who received corticosteroids and/or oxygen supplement were not significantly different between the two groups. The severity of COVID-19 in its acute phase was defined by the Ministry of Health, Labour and Welfare in Japan [24]. The percentage of moderate-severe cases was significantly larger in ME/CFS patients than in non-ME/CFS patients (40.4% vs. 21.6%; *p* = 0.006). The numbers (proportions) of ME/CFS patients with incomplete COVID-19 vaccination (who did not receive vaccination or received only one dose) and vaccination with at least two doses were 33 (70.2%) and 13 (27.7%), respectively. The number of patients with adequate vaccination (two or three doses) was not significantly different between the ME/CFS and non-ME/CFS groups. The median durations after the onset of COVID-19 to the first visit were 4.2 months in ME/CFS patients and 2.5 months in non-ME/CFS patients (*p* < 0.001).

**Figure 1:**
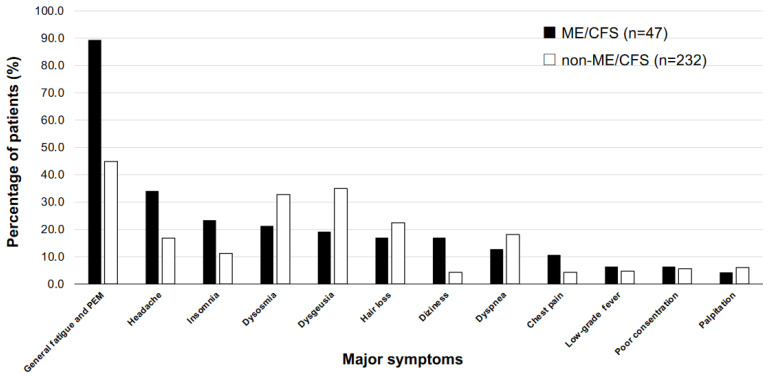
Clinical characteristics of long COVID patients who met and did not meet the criteria of ME/CFS. The percentages of major symptoms in ME/CFS patients and non-ME/CFS patients (ME/CFS: *n* = 47; non-ME/CFS: *n* = 232) are shown. PEM: post-exertional malaise.

The vital signs at the first visit in the ME/CFS patients and non-ME/CFS patients were not notably different (Table 3). The patient-reported questionnaires showed significantly higher FAS, significantly lower EQ-ED, significantly lower EQ-VAS and significantly higher SDS in the ME/CFS patients (Table 3). The percentages of symptoms in the ME/CFS patients and non-ME/CFS patients (ME/CFS: *n* = 47, non-ME/CFS: *n* = 232) are shown in Figure 1. The most frequent symptoms in ME/CFS patients were general fatigue and PEM (42 patients, 89.4%), headache (16 patients, 34.0%), insomnia (11 patients, 23.4%), dysosmia (10 patients, 21.3%) and dysgeusia (9 patients, 19.1%). On the other hand, the most frequent symptoms in non-ME/CFS patients were general fatigue and PEM (104 patients, 44.8%), dysgeusia (81 patients, 34.9%), dysosmia (76 patients, 32.8%) hair loss (52 patients, 22.4%) and dyspnea (42 patients, 18.1%).

**Figure 2:**
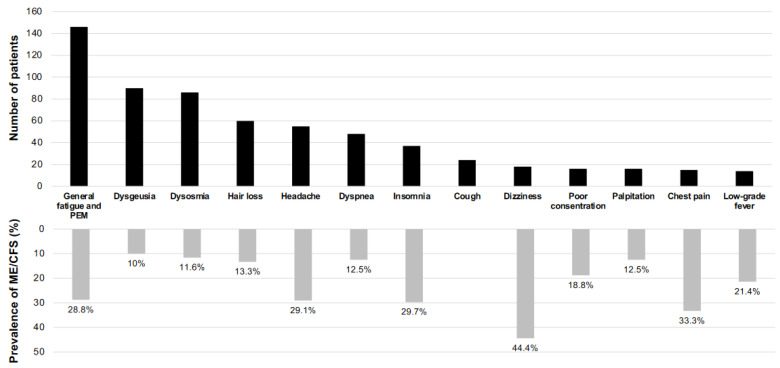
Relationships between major symptoms of long COVID and prevalence of ME/CFS. The upper panel shows the total number of major complaints in long COVID patients (*n* = 279) and in lower panel shows the symptom-based percentages of patients who met the criteria of ME/CFS.

The upper panel of Figure 2 shows the total number of complaints in post COVID-19 (*n* = 279). The symptoms of long COVID were varied in more than 10 symptoms, and the most frequent symptoms were general fatigue and PEM (146 patients, 52.3%), dysgeusia (90 patients, 32.3%), dysosmia (86 patients, 30.8%) and headache (55 patients, 19.7%). The lower panel of Figure 2 shows symptom-based percentages of patients with ME/CFS. The most frequent symptom in ME/CFS patients was dizziness. Eight (44%) of 18 long COVID patients who complained of dizziness met the ME/CFS criteria. Other symptoms were chest pain (in 15 patients including 5 patients with ME/CFS (33.3%)), insomnia (in 37 patients including 11 patients with ME/CFS (29.7%)), headache (in 55 patients including 16 patients with ME/CFS (29.1%)), and general fatigue and PEM (in 146 patients including 42 patients with ME/CFS (28.8%)).

## 4. Discussion

In the present study, we clarified the proportion and characteristics of long COVID patients who had ME/CFS patients in long COVID. To the best of our knowledge, this study is the first study in which ME/CFS was diagnosed in long COVID patients after face-to-face consultation and exclusion of diseases and the first study to show the proportion of long COVID patients with ME/CFS, which is a novel part of this study. In our study, the prevalence of ME/CFS was revealed to be 16.8%, which is close to the prevalences reported previously [8,18].

The results of this study showed that patients in the ME/CFS group had a longer time between the onset of COVID-19 and the first visit (4.2 months in the ME/CFS group vs. 2.5 months in the non-ME/CFS group as shown in Table 1) and had higher FAS (38 vs. 28, *p* < 0.0001), lower quality of life scores (0.6556 vs. 0.7840, *p* = 0.0001) and higher SDS (50 vs. 46, *p* = 0.0011) at the time of the first visit as shown in Table 2. Our earlier studies for patients visiting our CAC [6,25] and a study conducted at another institution [5] also showed that the most frequent complaint in patients immediately after the onset of COVID-19 was fatigue and that the percentage of patients complaining of fatigue decreased over time, suggesting that patients transitioning from long COVID to ME/CFS may have long-lasting symptoms.

Considering that the SDS scores were higher in ME/CFS patients at the first visit, it is likely that such patients already had a depressive mood at an earlier stage. However, SDS is a self-administered questionnaire, and it is possible that SDS was higher in ME/CFS patients because they had strong symptoms of general fatigue and post-exertional malaise. Since a six-month symptomatic period is required as a criterion for diagnosis of ME/CFS, it is possible that the duration after the onset of COVID-19 to the first visit to our CAC was also longer. Furthermore, more ME/CFS patients had a severe status in the COVID-19 acute phase (percentage of patients with moderate to severe status: 40.4% in ME/CFS patients vs. 21.6% in non-ME/CFS patients, *p* = 0.006). An association between long COVID and initial disease severity during the acute phase of COVID-19 was not found in most studies [5,8]. However, a few studies have shown that severe COVID-19 requiring invasive mechanical ventilation, intensive care unit (ICU) admission or prolonged hospitalization was associated with persistent symptoms [26,27].

The patients with ME/CFS in this study had fewer symptoms including dysosmia, dysgeusia and hair loss than did the non-ME/CFS patients at the initial visit to our CAC (Figure 1). This suggests that general fatigue and PEM are symptoms that are more important for ME/CFS than are other post COVID-19 condition symptoms. In fact, patients in the ME/CFS group had higher FAS at the first visit, which was similar results when selectively analyzed mental and physical fatigue. The gender ratio in the prevalence of ME/CFS caused by long COVID was approximately 1:1 in our study. This is a notable difference from previously reported results of epidemiology studies for the prevalence of ME/CFS (about 70–80% in women) [28,29].

Our study is also unique in that all of the patients received face-to-face examinations, which were not conducted in previous studies [29,30]. We were therefore able to rule out differential diseases with ME/CFS-like symptoms. For example, cases of isolated ACTH deficiency and adult GH deficiency mimicking ME/CFS have been reported [31], and it is critical to perform detailed history taking, physical examination and laboratory examination in order to exclude other somatic disease such as diabetes, hypothyroidism and rheumatological disease [32]. On the other hand, it is important to consider co-morbid entities [33] such as fibromyalgia, myofascial pain syndrome, irritable bowel syndrome and migraine [10]. For instance, LOH syndrome, a complication of ME/CFS, has also been reported, indicating the importance of investigating endocrine and metabolic abnormalities [34,35].

The lower panel of Figure 2 shows the key symptoms that are characteristic of ME/CFS. Dizziness, fatigue and PEM, headache, malaise and chest pain are all significant items in the ME/CFS diagnostic criteria [9,10,11]. However, complaints of these symptoms were made by less than 40% of the patients because information was obtained at the first visit to our CAC. The patients who met the ME/CFS criteria fulfilled the essential diagnostic items during their course. Some of the diagnostic criteria for ME/CFS include orthostatic dysregulation and it was thought that there might be a tendency for tachycardia [36], but the results of this study showed no statistically significant differences in these between the ME/CFS and non-ME/CFS patients (Table 3). Objective tests for orthostatic intolerance would be needed for further evaluation [37].

The present study has several limitations. First, this study was carried out to determine the prevalence of ME/CFS in post COVID-19 patients who visited an outpatient clinic that is a single center in Japan. Moreover, the backgrounds of patients were long COVID patients who were referred to our CAC, therefore it is possible that the patients suffering more severe and prolonged symptoms were included in this study. Second, we may not have adequately detected patients with ME/CFS, since the study included patients who had ME/CFS only a few months after the onset of ME/CFS, and there are patients in the non-ME/CFS group in whom ME/CFS might have developed in the future. Third, this study included only patients who were referred to a specific outpatient clinic and the entire long COVID or post COVID-19 population was not examined. Fourth, various strains of COVID-19 including Delta and Omicron strains have emerged, but the differences in symptoms due to these strains were not investigated in this study. Finally, changes in symptoms over time due to vaccination were not evaluated.

## 5. Conclusions

Collectively, we revealed the prevalence rate of ME/CFS and characteristic sequelae in patients with long COVID who visited our CAC, which is an outpatient clinic specializing in long COVID. Overall, 16.8% of long COVID patients met the ME/CFS criteria. Medical practitioners who care for patients with long COVID should also consider the possibility of ME/CFS in cases of prolonged symptoms including general fatigue and PEM. Further research is needed to determine the pathophysiology of ME/CFS and the possibility of medical and physical treatments.

## Figures and Tables

**Table 1:** Numbers and proportions of patients who met the ME/CFS criteria.

Types of Criteria	Number (%) of Patients (*n* = 279)
Fukuda, 1994	48 (17.2)
Canada, 2003	50 (17.9)
IOM, 2015	50 (17.9)
met all of the above criteria	47 (16.8)

IOM: Institute of Medicine.

**Table 2:** Backgrounds of ME/CFS and non-ME/CFS patients who visited the COVID-19 aftercare outpatient clinic.

	ME/CFS (*n* = 47)	Non-ME/CFS (*n* = 232)	*p*-Value
Age			
Total age	40 (30–52)	41 (27–90)	0.126
<19 years	5 (10.6%)	27 (11.6%)	
20–29 years	7 (14.9%)	39 (16.8%)	
30–39 years	10 (21.3%)	43 (18.5%)	
40–49 years	9 (19.1%)	56 (24.1%)	
50–59 years	11 (23.4%)	44 (19.0%)	
60–69 years	5 (10.6%)	13 (5.6%)	
>69 years	0 (0%)	10 (4.3%)	
Gender			0.4618
Male	23 (48.9%)	101 (43.5%)	
Female	24 (51.1%)	131 (56.5%)	
BMI			0.1211
Total BMI	24.7 (21.4–27.6)	23.0 (20.4–26.0)	
<25	26 (55.3%)	159 (68.5%)	
25–30	17 (36.2%)	54 (23.3%)	
>30	4 (8.5%)	19 (8.2%)	
Habits			
Smoking	23 (48.9%)	88 (37.9%)	0.16
Alcohol drinking	26 (55.3%)	111 (47.8%)	0.35
Acute phase status			
Admission	21 (44.7%)	64 (27.6%)	0.02 *
Use of steroids or oxygen	14 (29.8%)	48 (20.8%)	0.171
Severity of COVID-19 in acute phase			0.006 *
Mild	28 (59.6%)	182 (78.4%)	
Moderate-Severe	19 (40.4%)	50 (21.6%)	
COVID-19 vaccination status			0.111
Incomplete vaccination (0 or 1 dose)	33 (70.2%)	135 (58.2%)	
Vaccination with at least 2 doses	13 (27.7%)	93 (40.1%)	
unknown	1 (2.1%)	4 (1.7%)	
Duration after the onset of COVID-19 to the first visit			
Total duration (months)	4.2 (2.4–6.8)	2.5 (1.7–4.1)	0.0001 *
1–2 months	6 (12.8%)	75 (32.3%)	
2–3 months	8 (17.0%)	51 (22.0%)	
3–4 months	8 (17.0%)	35 (15.1%)	
4–5 months	7 (14.9%)	30 (12.9%)	
5–6 months	1 (2.1%)	12 (5.2%)	
>6 months	17 (36.2%)	29 (12.5%)	

Medians [IQR: interquartile ranges] and percentages (%) are shown. BMI: body mass index. We regarded * *p* < 0.05 as statistically significant differences between the two groups.

**Table 3:** Vital signs and patient-reported questionnaires of ME/CFS and non-ME/CFS patients who visited the COVID-19 aftercare outpatient clinic.

	ME/CFS	(Number)	Non-ME/CFS	(Number)	*p*-Value
SBP (mmHg)	129 (114–138)	(*n* = 47)	121 (108–136)	(*n* = 232)	0.0382 *
DBP (mmHg)	73 (64–81)	(*n* = 47)	71 (63–82)	(*n* = 232)	0.5760
PR (bpm)	82 (76–87)	(*n* = 47)	81 (73–90)	(*n* = 232)	0.7204
SpO2 (room air) (%)	98 (98–99)	(*n* = 47)	98 (98–99)	(*n* = 230)	0.1116
RR (/min)	18 (16–20)	(*n* = 46)	18 (16–20)	(*n* = 229)	0.7001
BT (℃)	36.7 (36.5–36.9)	(*n* = 46)	36.7 (36.5–36.9)	(*n* = 230)	0.551
FAS	38 (32–41)	(*n* = 47)	28 (19–35)	(*n* = 229)	<0.0001 *
FAS physical	21 (18–22)	(*n* = 47)	16 (12–19)	(*n* = 229)	<0.0001 *
FAS mental	17 (14–19)	(*n* = 47)	12 (7–16)	(*n* = 229)	<0.0001 *
EQ-5D	0.6556 (0.5331–0.7606)	(*n* = 44)	0.7840 (0.6558–0.8945)	(*n* = 220)	0.0001 *
EQ-5D VAS	50 (40–60)	(*n* = 45)	67.5 (50–80)	(*n* = 220)	0.0001 *
SDS	50 (45–56.5)	(*n* = 47)	46 (38–52)	(*n* = 224)	0.0011 *

Medians (IQR: interquartile ranges) are shown. SBP: systolic blood pressure, DBP: diastolic blood pressure, PR: pulse rate, SpO2: saturation of percutaneous oxygen, RR: respiratory rate, BT: body temperature, FAS; fatigue assessment scale, EQ-5D: EuroQol 5 dimensions 5-level, VAS: Visual Analog Scale, SDS: Self-rating Depression Scale. We regarded * *p* < 0.05 as statistically significant differences between the two groups.

## Data Availability

Detailed data will be available if requested to the corresponding author.

## Abbreviations

Body mass index (BMI); chronic fatigue syndrome (CFS); coronavirus disease 2019 (COVID-19); COVID-19 aftercare clinic (CAC); Fatigue Assessment Scale (FAS); myalgic encephalomyelitis (ME); post-exertional malaise (PEM); and Self-Rating Depression Scale (SDS).
